# 异基因造血干细胞移植后人细小病毒B19感染24例临床分析

**DOI:** 10.3760/cma.j.cn121090-20231128-00284

**Published:** 2024-06

**Authors:** 晋 张, 瑞 马, 雪宜 罗, 晓辉 张, 兰平 许, 昱 王, 晓东 莫, 萌 吕, 开彦 刘, 晓军 黄, 于谦 孙

**Affiliations:** 北京大学人民医院、北京大学血液病研究所、国家血液系统疾病临床医学研究中心，造血干细胞移植治疗血液病北京市重点实验室，北京 100044 Peking University People's Hospital, Peking University Institute of Hematology, National Clinical Research Center for Hematologic Disease, Beijing Key Laboratory of Hematopoietic Stem Cell Transplantation, Beijing 100044, China

## Abstract

人细小病毒B19（HPVB19）属细小病毒科，红细胞病毒属，与多种人类疾病相关，HPVB19感染是异基因造血干细胞移植（allo-HSCT）后难治性贫血的重要原因之一。此项研究对24例HSCT合并HPVB19感染的患者进行了回顾性分析，旨在整理总结allo-HSCT术后合并HPVB19感染患者的临床表现、治疗与转归，为allo-HSCT后HPVB19感染的管理提供经验。HPVB19感染患者中位年龄为25岁，感染发生的中位时间为移植后107 d（+107 d），有22例（91.7％）患者存在贫血，HGB中位数为77.5（46～149）g/L；新发贫血或血红蛋白持续下降的患者有13例（54.2％）。患者的中位住院时长为19 d，在新发贫血或HGB持续下降的患者中，经过静脉丙种球蛋白和（或）抗病毒治疗后HGB平均升高15.69 g/L，10例（76.92％）患者治疗有效。HSCT后出现难治性贫血时应警惕HPVB19感染；尽管缺乏特异性的治疗手段，HPVB19感染患者总体预后较好。

## Abstract (en)

Human parvovirus B19（HPVB19）belongs to Parvoviridae, a genus of erythrovirus, and has been associated with various human diseases, and HPVB19 infection is one of the most important causes of refractory anemia after allogeneic hematopoietic stem cell transplantation（allo-HSCT）. This study retrospectively analyzed 24 patients with HSCT combined with HPVB19 infection to collate and summarize the clinical presentation, treatment, and regression of patients with combined HPVB19 infection after allo-HSCT and provide experience in the management of HPVB19 infection after allo-HSCT. The median age of the patients with HPVB19 infection was 25 years, and the median time of infection occurrence was +107 days after transplantation, and 22（91.7％）had anemia with a median hemoglobin（HGB）level of 77.5（46–149）g/L, and 13（54.2％）had new-onset anemia or persistent decline in HGB. The median length of hospital stay was 19 days. Among patients with new-onset anemia or persistent decline in HGB, the mean increase in HGB after treatment with intravenous immunoglobulin and/or antiviral therapy was 15.69 g/L, and treatment was effective in 10（76.92％）patients. HPVB19 infection should be alerted to the development of refractory anemia after HSCT; despite the lack of specific treatment, the overall prognosis of HPVB19-infected patients is good.

人细小病毒B19（HPVB19）是一种细小病毒科红病毒属的单链DNA病毒，人群普遍易感，可导致多种人类疾病[1]。在免疫正常人群中，大部分HPVB19感染无明显症状，或仅有部分患者表现出不适、头痛、咽痛等流感样症状[2]。异基因造血干细胞移植（allo-HSCT）后患者由于抗病毒免疫功能严重受损，IgM和IgG产生速度慢或不产生导致患者没有中和病毒的能力，会导致慢性感染与长时间的病毒血症，移植后感染发生率为5.7％～30.0％[3]–[4]，而移植后患者感染HPVB19后贫血的发生率极高，部分患者可能发生纯红细胞再生障碍（PRCA）、甚至植入失败等严重并发症[5]，也有可能导致器官损伤如心肌炎、肝炎[6]、肺炎、急性胸膜心包炎、多器官衰竭、移植物抗宿主病[7]等，影响移植预后。

本研究我们回顾性分析了24例HSCT后合并HPVB19感染患者的临床特点、治疗效果及转归，以期提高临床医师对HPVB19相关疾病的认识，为allo-HSCT后HPVB19感染的管理提供借鉴。

## 病例与方法

1. 病例：本研究为回顾性研究，纳入2016年1月1日至2022年11月13日在北京大学人民医院接受首次allo-HSCT且移植后通过PCR核酸检测确诊HPVB19感染的患者24例。本研究经北京大学人民医院伦理委员会批准（批件号：2023PHB336-001）。

2. 造血干细胞移植方案：HLA全相合移植2例（8.3％），单倍体移植22例（91.7％）。供者为父母15例（62.5％），供者为同胞6例（25.0％），供者为子女3例（12.5％）。ABO血型相合11例（45.8％），主要不合6例（25％），次要不合5例（20.8％），主次均不合2例（8.3％）；8例（33.3％）患者移植物为外周血造血干细胞，15例（62.5％）为骨髓+外周血造血干细胞，1例（4.2％）为脐带血。患者回输MNC中位数为9.97（4.99～17.02）×10^8^/kg，CD34^+^中位数为2.90（1.14～9.23）×10^6^/kg。具体移植方案见文献[8]。所有患者均接受环孢素A（CsA）+短程甲氨蝶呤（MTX）联合霉酚酸酯（MMF）预防移植物抗宿主病（GVHD）。

3. HPVB19筛查及诊断标准：当患者出现移植后原因不明的贫血或发热时进行HPVB19筛查。采用人细小病毒实时荧光定量PCR试剂盒（上海之江生物科技有限公司）检测HPVB19 DNA，试剂盒检测下限为1×10^3^拷贝数/ml，当患者外周血PCR检测结果大于检测下限时判定为阳性。

4. 统计学处理：本研究连续资料不服从正态分布，因此采用中位数（范围）进行统计描述，分类资料则采用例数（构成比）进行统计描述。

## 结果

一、患者基本信息

24例患者中，男15例（62.5％），女9例（37.5％），中位年龄25（6～59）岁。原发病包括急性淋巴细胞白血病（ALL）9例（37.5％）、急性髓系白血病（AML）5例（20.8％）、骨髓增生异常综合征（MDS）4例（16.7％）、再生障碍性贫血（AA）与慢性髓性白血病（CML）各2例（8.3％）、髓系肉瘤与T淋巴母细胞淋巴瘤（T-LBL）各1例（4.2％）。24例患者中性粒细胞中位植活时间为13（10～26）d；1例患者血小板未植活，其余23例患者血小板植活中位时间为14.5（9～16）d。24例患者中5例（20.8％）发生急性GVHD（aGVHD）；5例（20.8％）患者合并巨细胞病毒（CMV）感染，中位感染时间为38（22～91）d；4例（16.7％）患者合并EB病毒（EBV）感染，中位感染时间为+43（+24～+51）d。

二、临床表现

HPVB19感染发生的中位时间为+107（+47～+1321）d，其中83.3％（20/24）的感染在移植后5个月内发生，3例患者HPVB19血症发生时间超过移植后1年。

1. 血液学表现：24例患者中，22例（91.7％）存在贫血，HGB中位数为77.5（46～149）g/L；新发贫血或HGB持续下降的患者有13例（54.2％）。8例（33.3％）患者存在中性粒细胞减少，ANC中位数为1.68（0.60～9.89）×10^9^/L，其中较确诊感染前最近1次血常规检查出现粒细胞减少或粒细胞缺乏者有8例（33.3％）。20例（83.3％）患者存在血小板减少，PLT中位数为50（4～403）×10^9^/L，PLT较确诊感染前最近1次血常规检查下降>30％或出现血小板输注依赖者有8例（33.3％）。

2. 骨髓表现：9例患者进行了骨髓穿刺检查，其中有3例存在PRCA，2例存在粒系造血受抑，1例表现为红系增生活跃，其余3例骨髓检验结果正常。3例PRCA患者均为感染后新发骨髓原红细胞减少，且经过治疗后分别于1、2、4个月后复查骨髓象时恢复正常。

3. 其他表现：12例（50.0％）患者伴有发热，3例出现了新发皮疹，1例出现了新发转子滑囊炎。

三、治疗及转归

共21例（87.5％）患者在接受了静脉注射免疫球蛋白（IVIG）治疗，中位使用剂量为20（5～20）g/d，（其中11例使用剂量均为20 g/d），中位使用疗程为5（1～10）d，使用IVIG的中位总剂量为80（10～200）g。14例（58.3％）患者同时接受了抗病毒治疗，其中8例使用阿昔洛韦作为抗病毒药物，其余患者使用更昔洛韦、膦甲酸钠等。中位治疗时长为19（11～32）d，2例患者死亡，死亡原因分别为重度GVHD与持续重症感染，均与HPVB19无关，1例患者自行离院，其余21例均病情平稳。

在出现新发贫血或HGB持续下降的HPVB19感染患者中，经过治疗后HGB平均升高（15.69±19.85）g/L，较确诊时HGB平均数升高25.34％，10例（76.92％）患者HGB较确诊时升高，7例（53.85％）患者HGB恢复至感染前最后1次血常规检查水平。24例患者中，7例患者出院时病毒DNA检测转阴，中位转阴时间为12（5～82）d；3例（12.5％）患者在1年内出现HPVB19复发感染，再次出现DNA检测阳性的时间分别为转阴后8、41、169 d。1例患者表现为HPVB19超过3个月DNA检测弱阳性，IVIG治疗不能清除细小病毒血症。

## 讨论

既往研究报道，allo-HSCT后HPVB19感染的总体发生率为5.7％～30.0％。发生病毒血症的中位时间为移植后1～5个月不等[3],[6],[9]。本组资料显示，HPVB19 DNA检测阳性集中于移植后后2～5个月发生，与以往研究吻合[5]，提示在移植后应关注HPVB19感染的可能性。

HPVB19的感染是导致移植后PRCA的主要原因之一。既往研究显示移植后合并HPVB19感染的患者中贫血的发生率为99％，同时，粒细胞减少症与血小板减少症也有38％与21％的发生率[10]。本研究中，贫血的发生率为91.7％，33.3％的患者较确诊感染前最近1次血常规检查出现粒细胞减少或粒细胞缺乏，33.3％的患者PLT较确诊感染前最近1次血常规检查下降>30％或出现血小板输注依赖，与既往研究大致相符。目前主要观点认为HPVB19感染后的血小板减少与骨髓抑制与免疫介导有关，即发病早期NS1蛋白可能介导对巨核细胞的细胞毒效应致使血小板生成减少，而发病晚期病毒感染介导免疫系统产生抗血小板抗体导致血小板被过度清除[11]。HPVB19感染相关的粒细胞减少症也偶见报道，其具体机制至今尚不明确，Shirono等[12]报道了7例HPVB19相关的病毒相关性噬血细胞综合征（VAHS），患者可表现为全血细胞减少，作者推测其背后机制或许能解释原因不明的HPVB19感染后粒细胞减少。Mende等[13]报道骨髓检查中HPVB19感染的特异性表现为红系造血减少，嗜碱性和空泡性细胞质、染色质不凝结的巨大原红细胞，核内有紫色的病毒包涵体。但在本研究收集到的骨髓穿刺样本中均未观察到包涵体。从可获得的9例骨髓穿刺结果来看，HPVB19感染后骨髓检查结果个体差异大，贫血的程度与红系造血活跃程度之间没有明显的关系，这一现象值得我们进一步探索和验证；但值得注意，本研究中得到的骨髓穿刺结果均为细小病毒抗体阳性期间获得，并不能确定穿刺时间与感染发生时间的关系，且病例数较少，以上均可能是贫血与骨髓造血之间缺乏相关性的原因。

移植后发生的HPVB19感染尚且没有统一的治疗方案，由于缺乏特异性的抗病毒药物，临床常用IVIG 400 mg·kg^−1^·d^−1^连续5 d，通常在10 d内可以看到网织红细胞计数回升[3]。本组24例患者中，76.92％的患者HGB较确诊时回升，53.85％的患者HGB恢复至感染前最后1次血常规检查水平，这提示了IVIG治疗的有效性。必须注意到本研究存在一些局限性，如未检测网织红细胞计数，而是采取连续监测HGB水平来判断治疗效果；本文样本量少，个体差异大，大部分仅呈现个体的特征，由本案例报道得出的结论可能存在偏差。

综上所述，本研究提示HPVB19感染是移植后难治性贫血的可能病因之一，经过IVIG治疗，HPVB19感染患者总体预后较好。但本研究病例数较少，需进一步研究探索。
